# High Consumption of Ultra-Processed Foods Is Associated with Genome-Wide DNA Methylation Differences in Women: A Pilot Study

**DOI:** 10.3390/nu17213465

**Published:** 2025-11-03

**Authors:** Alessandra Escorcio Rodrigues, Ariana Ester Fernandes, Alexis Germán Murillo Carrasco, Felipe Mateus Pellenz, Paula Waki Lopes da Rosa, Aline Maria da Silva Hourneaux de Moura, Fernanda Galvão de Oliveira Santin, Cintia Cercato, Maria Edna de Melo, Marcio C. Mancini

**Affiliations:** 1 Grupo de Obesidade e Sindrome Metabolica, Hospital das Clinicas, HCFMUSP, Faculdade de Medicina, Universidade de Sao Paulo, Sao Paulo, SP 05403-000, Brazil; ariana@usp.br (A.E.F.); maria.edna@hc.fm.usp.br (M.E.d.M.); mmancini@usp.br (M.C.M.); 2 Center for Translational Research in Oncology, Instituto do Cancer do Estado de São Paulo, Faculdade de Medicina, Universidade de Sao Paulo, Sao Paulo, SP 01246-903, Brazil; agmurilloc@usp.br; 3 Comprehensive Center for Precision Oncology, Universidade de Sao Paulo, Sao Paulo, SP 01246-000, Brazil; 4 Escola da Saude, Universidade do Vale do Rio dos Sinos-UNISINOS, São Leopoldo, RS 93022-750, Brazil; felpellenz@unisinos.br; 5 Department of Nutrition, Faculdade de Saude Publica, Universidade de São Paulo, Sao Paulo, SP 01246-904, Brazil

**Keywords:** ultra-processed foods, DNA methylation, epigenetics, next-generation sequencing, diet quality

## Abstract

**Background/Objectives:** The global increase in the consumption of ultra-processed foods (UPFs) parallels the rise in obesity and non-communicable chronic diseases. Although several large-scale studies associate UPF intake with adverse health outcomes, the biological mechanisms remain unclear. Epigenetic alterations, such as changes in DNA methylation, may represent a potential pathway by which diet influences metabolic health. The aim of this study was to investigate whether higher UPF consumption is associated with genome-wide DNA methylation patterns in women. **Methods:** This was a cross-sectional observational study with exploratory epigenetic analysis. We selected 30 women, who were divided into tertiles based on their UPF consumption (expressed as a percentage of total energy intake) according to the NOVA food classification system. Dietary intake was assessed using a three-day food record. Anthropometric data, body composition and laboratory parameters were evaluated. The analysis of DNA methylation was performed utilizing DNA extracted from peripheral blood leukocytes of participants in the first and third tertiles of UPF consumption. Genome-wide methylation patterns were performed using next-generation sequencing. **Results:** Participants had a median (IQR) age of 31 years (26.0–36.5) and a BMI of 24.7 (23.6–35.8) kg/m^2^. For the epigenetic analyses, 15 women were included. Of the 30 women initially evaluated, 20 were included as they belonged to the first and third tertile of UPF consumption. Of these, five were excluded due to a low number of reads obtained by NGS. A total of 80 differentially methylated regions were identified between groups, most of which were hypomethylated in the high-UPF-intake group. **Conclusions:** High UPF consumption was associated with altered DNA methylation patterns, suggesting a potential epigenetic mechanism underlying the negative health effects of UPFs. This pilot study provides a model for future research with larger samples.

## 1. Introduction

Ultra-processed foods (UPFs), as established by the NOVA food classification system, comprise many ready-to-eat compounds, including soft drinks, packaged snacks, and ready-made meals. These products are described as industrial formulations made with little or no whole foods and typically contain chemically modified elements obtained from foods or synthesized in laboratories, in conjunction with added ingredients, such as colorings, flavorings, thickeners, and preservatives to enhance palatability, presentation, consistency, and durability. Their processing often involves techniques not commonly used in home cooking, such as extrusion or molding, with the goal of creating highly palatable, ready-to-eat or ready-to-heat products with an extended shelf life [1,2].

The classification of foods according to their degree of processing was proposed by Monteiro et al. and is currently adopted as a dietary guideline in several countries worldwide. The NOVA food classification system categorizes foods as follows: Group 1 includes unprocessed or minimally processed foods; Group 2 comprises culinary ingredients; Group 3 consists of processed foods, typically containing two or three ingredients—a combination of Group 1 and Group 2 foods; and Group 4 refers to UPF [1,2].

The consumption of UPF has increased worldwide in parallel with the rise in obesity and non-communicable chronic diseases, although substantial variation occurs within and between countries and regions [3,4,5,6]. In high-income countries, the dietary energy derived from UPF reaches half or more of the total energy intake (TEI), while in low- and middle-income countries, less than a third of caloric intake comes from these foods [6].

The escalating replacement of unprocessed and minimally processed foods by UPFs raises concerns about diet quality and population health, because these foods have poor nutrient profiles including low levels of micronutrients, vitamins, and fiber, as well as high calorie contents, added sugar, salt, trans and saturated fats. Moreover, UPFs often present changes in food matrices, food consistency, potential contaminants from ingredients of the package and manipulation, and presence of additives, sweeteners and other manufacturing ingredients [7].

A recently published randomized, unblinded, controlled crossover study investigated the effects of consumption of UPFs on body weight, energy intake and metabolism. Participants gained significantly more weight and consumed a significantly higher number of calories during the 1-week UPF period [8].

Emerging evidence indicates that higher UPF consumption is also associated with accelerated biological aging, independent of overall diet quality and likely driven by non-nutrient characteristics of these foods [9,10].

Large-scale, population-based studies have associated high UPF consumption with adverse health outcomes such as cardiovascular, respiratory, and gastrointestinal diseases; diabetes; obesity; anxiety, depression, and other mental disorders; and increased all-cause mortality [7,11]. Although this relationship is well documented, the biological mechanisms by which UPF consumption contribute to disease development remain unclear. More research is essential to understand the specific features that connect UPF to these adverse health outcomes.

One proposed mechanism involves epigenetic modifications, particularly changes in DNA methylation [12]. DNA methylation is an epigenetic mechanism in which a methyl group is added to cytosine residues at CpG sites through the action of DNA methyltransferases. This modification can alter gene expression without changing the underlying DNA sequence. CpG sites are often clustered in regions called CpG islands, which are frequently located near gene promoters. Methylation can affect gene expression directly, by blocking the binding of transcription factors and preventing transcription initiation, or indirectly, by recruiting proteins that bind to methylated DNA and regulate gene expression [13]. A recent study has demonstrated that DNA methylation patterns are influenced by overall dietary quality and even by specific nutrients, potentially affecting metabolic health [12]. In addition, a cross-sectional study reported that consumption of industrialized foods was associated with NR3C1 gene methylation [14]. However, studies that have correlated dietary patterns, particularly those considering the consumption of UPF, with DNA methylation are still scarce. Based on this knowledge gap, we hypothesized that UPF intake may influence epigenome-wide DNA methylation patterns, thereby impacting health.

To investigate this hypothesis, we conducted a cross-sectional exploratory study aimed at evaluating whether women with higher UPF consumption exhibit different genome-wide DNA methylation patterns compared to those with lower UPF consumption.

## 2. Materials and Methods

### 2.1. Study Design and Participants

We selected 30 women from a previous study conducted by our group [15]. The inclusion criteria were as follows: eumenorrheic women aged 20 to 40 years, with a BMI ranging from 18.5 to 39.9 kg/m^2^. The exclusion criteria were as follows: participants with eating disorders, comorbid conditions, over-exercising, amenorrhea, infectious disease, cancer, pregnancy, breastfeeding, use of medications that affect body weight or eating behavior, smoking, and the presence of active alcohol dependence or drug addiction. The study was approved by the Ethics Committee for Analysis of Research Projects of the Hospital das Clínicas of the University of Sao Paulo, Brazil (CAAE: 57631816300000068, issued on 20 April 2017), and all participants provided written informed consent prior to participation, in accordance with the ethical standards of the Declaration of Helsinki.

### 2.2. UPF Consumption

Participants were divided into tertiles according to UPF consumption, expressed as a percentage of total daily energy intake. Dietary intake was assessed using a three-day food record (two non-consecutive weekdays and one weekend day) [16] and analyzed with Avanutri^®^ software (version 4.0), using the Brazilian Food Composition Table–TACO [17] as reference. The analysis included mean absolute intake of energy (kcal), cholesterol (mg), dietary fiber (g), and the percentage contribution of macronutrients (carbohydrates, proteins, total fats) and fat subtypes (saturated, monounsaturated, polyunsaturated). Foods were classified by degree of processing according to the NOVA system [1,2]. Total energy intake for each NOVA category was adjusted for habitual intake using the Multiple Source Method to account for intra-individual variability [18].

### 2.3. Collect of Anthropometric Information

Anthropometric data (weight, height, and waist circumference), body composition (estimated by bioelectrical impedance analysis—InBody 720, Biospace Co., Ltd., Seoul, Republic of Korea) and laboratory parameters (aspartate aminotransferase, alanine aminotransferase, gamma-glutamyl transferase, glucose, insulin, glycated hemoglobin, total cholesterol, HDL cholesterol, LDL cholesterol, non-HDL cholesterol, triglyceride, leptin, and adiponectin levels) were obtained.

### 2.4. DNA Extraction

DNA was extracted from peripheral blood leukocytes. The DNA samples were subjected to genome-wide methylation profiling, which was performed using next-generation sequencing (NGS) with methylated DNA enrichment (MethylMiner™, Thermo Fisher Scientific, Waltham, MA, USA) and bisulfite conversion. The enrichment step was conducted after enzymatic fragmentation and library preparation to prevent bisulfite-induced DNA degradation and to enhance detection sensitivity [19].

### 2.5. Methylome Analyses

Sequencing was performed on the Ion Chef™ platform using the Ion 520™ & Ion 530™ ExT Kit-Chef (Thermo Fisher Scientific Inc., Waltham, MA, USA) [20]. Sequencing reads, in FASTQ format, were processed using fastp software [21]. Paired-end alignment to the human reference genome (hg38) was performed using minimap2 [22]. Alignment files were sorted by genomic coordinates, and duplicate reads were removed using bamsort and bammarkduplicates from the biobambam2 suite [23].

Differential methylation analyses were conducted in R using packages from the Bioconductor repository [24]. Differentially methylated regions (DMRs) were identified using the MEDIPS package and annotated with ChIPpeakAnno [25]. Methylation intensity data were exported as BigWig files using the GenomicAlignments package, and Pearson correlation was used to assess co-methylation patterns [26].

### 2.6. Statistical Analyses

Statistical analyses of anthropometric, biochemical, and dietary intake data included the Shapiro–Wilk test to assess normality. Variables with normal distribution were expressed as mean ± standard deviation and compared using Student’s *t*-test. Non-normally distributed variables were presented as median (interquartile range) and compared using the Mann–Whitney U test. Statistical significance was set at *p* < 0.05.

Correlations among methylated regions were assessed using Pearson’s correlation test. To avoid excessive loss of power in this small exploratory pilot study (group 1: *n* = 7; group 2: *n* = 8), *p*-values are presented as nominal, without adjustment for multiple testing.

For data visualization, heatmaps were generated using the superheat package in R (version 4.4.2) [27]. Only regions with fold change > 4 were displayed.

## 3. Results

After analyzing dietary intake data and classifying participants into tertiles based on their UPF consumption, women in the first tertile (consuming a mean of 14% of total daily energy intake from UPF) and in the third tertile (mean of 45%) were selected to have their DNA samples subjected to NGS. Of the 20 samples subjected to NGS, 5 generated fewer than 1,000,000 reads in the NGS analysis and were excluded from the study before statistical and bioinformatic analyses. The final dataset included 15 samples: 7 from the low-UPF-consumption group and 8 from the high-UPF-consumption group.

Our sample consisted of 15 women with a median (IQR) age of 31 years (26–36.5) and a BMI of 24.7 (23.6–35.8) kg/m^2^. Most participants self-identified as White 66.7%, while 20% self-identified as Black and 13.3% as Mixed Race. A total of 93% of participants had some level of post-secondary education (including incomplete and complete college, or postgraduate degree), while 10% had only primary or secondary education.

Comparison of anthropometric and body composition data (including age, weight, height, BMI, waist circumference, waist–height ratio, lean mass, fat-free mass, and fat mass) revealed no significant differences between groups (Table 1).

Regarding laboratory parameters, total cholesterol, LDL cholesterol and non-HDL cholesterol were higher in the low-UPF-intake group (*p* = 0.005, 0.001, and 0.002, respectively) (Table 1).

Comparison of the dietary data showed that the consumption of UPFs (% of TEI) in the low-UPF group was 14.3% ± 5.8% and in the high-UPF group 45.1% ± 2.8% (*p* < 0.001). Reciprocally, unprocessed/minimally processed food consumption was higher in the low-UPF group compared to the high-UPF group (61.4% ± 10.4% vs. 39.7% ± 5.4%, *p* < 0.001). Furthermore, the low-UPF group had higher percentage of protein intake (*p* = 0.013) and polyunsaturated fat intake (*p* = 0.034) (Table 2).

**Figure 1:**
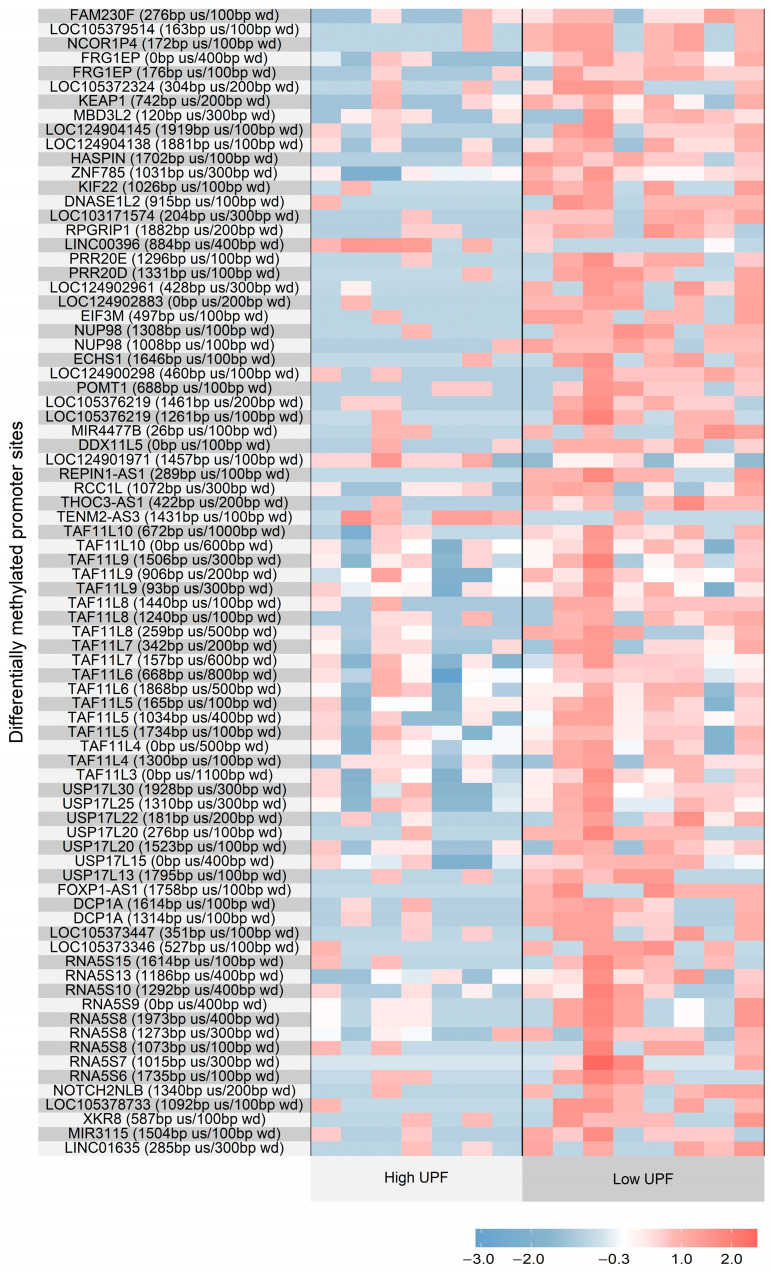
Heatmap of differentially methylated promoter regions comparison between women with high and low ultra-processed food intake. Samples were grouped into two categories based on ultra-processed food (UPF) consumption: low consumption and high consumption. Each row represents a promoter region associated with a gene, identified by its symbol, the distance in nucleotides to the transcription start site (upstream—us), and the total width of the analyzed region (width—wd). Columns represent individual samples, grouped according to higher or lower UPF consumption. Color intensity ranges from blue (hypomethylation) to red (hypermethylation), based on standardized methylation values (z-scores).

We identified 80 DMRs between groups, primarily in gene promoter regions (nominal *p* < 0.05). Applying the filter to the heatmap to highlight only regions where the difference was greater than fourfold revealed that the regions LINC00396, LOC124902961, REPIN1-AS1, FOXP1-AS1, RNA5S13, RNA5S9, and RNA5S7 exhibited the largest methylation differences between the groups. In the high-UPF group, most DMRs were hypomethylated. These promoter-associated DMRs are illustrated in Figure 1, with detailed correlation data provided in the Supplementary Material.

## 4. Discussion

This exploratory study is the first to use NGS to investigate genome-wide DNA methylation differences according to UPF intake in adults. This high-resolution approach revealed 80 DMRs between individuals with high and low UPF consumption. Most of these regions were hypomethylated in the high-UPF-consumption group, pointing to potential epigenetic mechanisms linking UPF intake to health outcomes.

The regions that stood out after applying the filter aimed at identifying the largest methylation differences (greater than fourfold) were LINC00396, LOC124902961, REPIN1-AS1, FOXP1-AS1, RNA5S13, RNA5S9, and RNA5S7. These regions exhibited the greatest methylation differences between the groups, being hypomethylated in the high-UPF-consumption group. Among them, REPIN1-AS1 and FOXP1-AS1 have received particular attention in the literature.

The REPIN1-AS1 transcript may regulate the REPIN1 gene and has been suggested as a predictor of gastric cancer progression [28]. REPIN1 overexpression appears to be associated with the development of NAFLD42 and plays a role in glucose and lipid metabolism by modulating the expression of its target genes, including glucose and fatty acid transporters, thereby influencing body fat distribution and insulin sensitivity [29,30,31,32]. FOXP1-AS1, when overexpressed, appears to be associated with breast cancer and lymphomas. This gene also appears to regulate the FOXP1 gene, which has been linked to autism and hematologic cancers [33,34,35].

Further exploratory studies are needed to confirm the associations between these genes and clinical outcomes.

The group with lower UPF consumption showed higher intake of protein and polyunsaturated fat, which are generally considered markers of diet quality and are commonly associated with greater consumption of unprocessed and minimally processed foods [1,2]. Several nutrients, including folate, B-complex vitamins, choline, polyphenols, and polyunsaturated fatty acids, are key regulators of DNA methylation. In vitro studies further confirm that docosahexaenoic acid, a polyunsaturated fatty, can induce differential methylation of CpG loci, with zinc-finger transcription factors potentially mediating the targeting of these epigenetic changes [36].

More recently, research has shifted toward dietary patterns, such as the Plant-Based and Mediterranean diets, which better capture eating behaviors and nutrient interactions and have been related to favorable epigenetic profiles [12]. These findings reinforce the importance of investigating the influence of dietary patterns on DNA methylation, as studies linking DNA methylation with overall diet quality remain scarce.

Unexpectedly, in this study, LDL cholesterol levels were higher in the low-UPF group (Table 2). Similarly, a recent crossover randomized a controlled feeding trial with two 8-week ad libitum diets following the UK Eatwell Guide, comparing a minimally processed food diet with an UPF diet. LDL cholesterol levels were found to be lower in the UPF diet. Further research is needed to better understand this [37].

A recent epigenome-wide association study (EWAS) also examined the relationship between UPF consumption and DNA methylation in children, identifying suggestive associations involving genes such as PHYHIP, NHEJ1, and ATF7, which are implicated in thyroid hormone signaling, liver function, and cancer-related pathways [38]. These findings support a potential epigenetic impact of UPF intake beginning in early life. However, the study acknowledged limitations in dietary assessment accuracy. In contrast, our study employed three-day food records, a more precise method compared to the single 24 h recall or food frequency questionnaires used in most pediatric cohorts [39]. Moreover, our dietary data were analyzed using the Multiple Source Method, which improves the estimation of habitual intake for both nutrients and food groups [18].

Furthermore, a relevant methodological distinction between our study and the aforementioned work lies in the DNA methylation assessment approach. While the previous study employed an EWAS using array-based technology to examine specific, predefined CpG sites, our study used NGS with methylated DNA enrichment and bisulfite conversion, enabling a more comprehensive, unbiased exploration of methylation patterns across the genome. Although EWAS platforms such as the Illumina 450 K or EPIC BeadChip offer good reproducibility and are suitable for large-scale studies, they are limited to a fixed set of CpG loci, often missing regulatory regions not included in the array design. In contrast, the NGS-based approach allowed the detection of novel DMRs, including those in promoter areas, thus providing broader insights into potential epigenetic mechanisms associated with UPF consumption [40,41].

This study has several strengths, including the use of high-resolution next-generation sequencing combined with methylated DNA enrichment and bisulfite conversion, allowing an unbiased genome-wide assessment of DNA methylation patterns. Additional strengths include the comprehensive dietary assessment using three-day food records analyzed through the Multiple Source Method, and the integration of advanced bioinformatic analyses to identify promoter-associated DMRs. However, some limitations should be acknowledged. The relatively small sample size may have reduced statistical power, and the cross-sectional design precludes causal inference. Moreover, because DNA methylation is tissue-specific, the patterns observed in peripheral blood leukocytes may not fully reflect those in other metabolically relevant tissues. Another limitation is the lack of adjustment for potential confounders, such as BMI and age, due to the small sample size; however, both groups had similar mean BMIs and age at baseline, which likely minimized potential confounding effects. Given that this was a pilot study with a limited sample and cross-sectional design, causal inference was not feasible. Future studies with larger cohorts could apply Mendelian randomization approaches to better explore potential causal relationships. Finally, the absence of detailed micronutrient intake data represents an additional limitation, as nutrients involved in one-carbon metabolism—such as folate and vitamin B12—can influence DNA methylation patterns.

## 5. Conclusions

High UPF consumption was associated with altered DNA methylation patterns, predominantly characterized by decreased methylation in specific genomic regions. These findings suggest a potential epigenetic mechanism through which UPF may contribute to adverse health effects. As a pilot study, this work provides a foundation for future research in larger cohorts.

## Figures and Tables

**Table 1:** Comparison of body composition, anthropometric and laboratory parameters between women with high and low ultra-processed food intake.

Variable	Low-UPF Group (*n* = 7)	High-UPF Group (*n* = 8)	*p*-Value
Age (years)	29 (27–34)	36 (24–37)	0.954
Weight (kg)	75.0 ± 19	75.5 ± 21.4	0.962
BMI (kg/m^2^)	28.9 (23.7–36.5)	24.7 (23–35.2)	0.779
Waist circumference (cm)	90.7 ± 17.3	91.4 ± 21.6	0.947
Lean mass (kg)	40.5 ± 5.2	41.8 ± 4.7	0.639
Fat mass (%)	40.5 ± 9.5	38.8 ± 10.7	0.757
Glucose (mg/dL)	77 ± 5.4	79.3 ± 9.0	0.555
Insulin (μIU/mL)	13.1 ± 7.5	14.1 ± 8.7	0.817
Glycated hemoglobin (%)	5.3 ± 0.3	5.3 ± 0.5	0.952
Total cholesterol (mg/dL)	195 ± 35	144.7 ± 21.5	0.005
LDL cholesterol (mg/dL)	119.6 ± 28.6	68.9 ± 14.9	0.001
HDL cholesterol (mg/dL)	56.8 ± 11.6	59.9 ± 15.3	0.670
Non-HDL cholesterol (mg/dL)	138.3 ± 33	84.9 ± 17.3	0.002
Triglycerides (mg/dL)	85.6 ± 34.5	82.3 ± 40.6	0.882
Aspartate aminotransferase (U/L)	17.3 ± 4.5	15.9 ± 5.6	0.61
Alanine aminotransferase (U/L)	15.6 ± 2.6	16,6 ± 7.0	0.745
Gamma-glutamyl transferase (U/L)	16.3 ± 6.1	16.3 ± 8,3	0.993
Adiponectin (μg/mL)	4.8 ± 2.8	6.5 ± 4.9	0.452
Leptin (ng/mL)	10.5 (8.0–23.9)	11.5 (6.4–31.6)	0.955

Parametric variables are presented as mean ± standard deviation (SD) and were analyzed using Student’s *t*-test; non-parametric variables are presented as median (IQR) and were analyzed using the Mann–Whitney test. UPF—ultra-processed food. BMI—body mass index, LDL—Low-Density Lipoprotein, HDL—High-Density Lipoprotein.

**Table 2:** Dietary assessment and comparison between women with high and low ultra-processed food intake.

Variable	Low-UPF Group (*n* = 7)	High-UPF Group (*n* = 8)	*p*-Value
Total energy intake (TEI)	1469 (1236–1645)	1391 (1234–1729)	0.955
Protein (% of TEI)	21.9 ± 5.0	15.2 ± 4.0	0.013
Carbohydrate (% of TEI)	40.5 ± 7.5	47.8 ± 7.3	0.08
Total fat (% of TEI)	36.9 ± 6.8	33.5 ± 5	0.294
Cholesterol (mg)	308.4 ± 133.4	207.8 ± 58	0.082
Saturated fat (% of TEI)	11.6 ± 1.9	9.4 ± 2.0	0.053
Polyunsaturated fat (% of TEI)	8.7 ± 2.2	6.5 ± 1.2	0.034
Monounsaturated fat (% of TEI)	10.5 ± 2.3	9.2 ± 3.1	0.352
Fiber (g)	13.3 (11–17.6)	7.1 (6.4–14.7)	0.463
Unprocessed/minimally processed foods (% of TEI)	61.4 ± 10.4	39.7 ± 5.4	<0.001
Culinary ingredients (% of TEI)	7.3 ± 4.4	6.5 ± 1.5	0.646
Processed foods (% of TEI)	10.3 ± 4.2	10.3 ± 6.8	0.994
Ultra-processed foods (% of TEI)	14.3 ± 5.8	45.1 ± 2.8	<0.001

Parametric variables are presented as mean ± standard deviation (SD) and were analyzed using Student’s *t*-test; non-parametric variables are presented as median (IQR) and were analyzed using the Mann–Whitney test. UPF—ultra-processed food, TEI—Total energy intake.

## Data Availability

The original contributions presented in this study are included in the article. Further inquiries can be directed to the corresponding author.

## Supplementary Materials

The following supporting information can be downloaded at: https://www.mdpi.com/article/10.3390/nu17213465/s1, Table S1: Differentially methylated regions (DMRs) identified between low- and high-UPF-consumption groups.

## Abbreviations

The following abbreviations are used in this manuscript:

UPF	Ultra-processed food
TEI	Total energy intake
NGS	Next-generation sequencing
IQR	Interquartile Range
DMRs	Differentially methylated regions
