# Supplementary material for: High Consumption of Ultra-Processed Foods Is Associated with Genome-Wide DNA Methylation Differences in Women: A Pilot Study
Source: Nutrients. 2025 Nov 3;17(21):3465. doi: 10.3390/nu17213465 (PMC12609220; doi:10.3390/nu17213465)
Supplement: Supplementary file 1 [file nutrients-17-03465-s001.zip › nutrients-3900108-supplementary.pdf]

Supplementary

Table S1 - Differentially methylated regions (DMRs) identified between low and high UPF consumption groups.

Region	seqnames	start	end	width	Low UPF.counts. mean	High UPF.counts. mean	edgeR.log FC	edgeR.p.valu e	Inside Feature	Distance To Site	symbol
RNA5S7 (1015bp us/300bp wd)	chr1	228624801	228625100	300	1,333333	0	3,613971	0,026555258	upstream	1015	RNA5S7
REPIN1-AS1 (289bp us/100bp wd)	chr7	150372201	150372300	100	1,25	0	3,608477	0,002563601	inside	289	REPIN1-AS1
FOXP1-AS1 (1758bp us/100bp wd)	chr3	71287901	71288000	100	1,25	0	3,564032	0,005287121	upstream	1758	FOXP1-AS1
RNA5S8 (1973bp us/400bp wd)	chr1	228628001	228628400	400	2,25	0,17857143	3,386301	0,008889388	upstream	1973	RNA5S8
RNA5S9 (0bp us/400bp wd)	chr1	228628001	228628400	400	2,25	0,17857143	3,386301	0,008889388	includeFeature	0	RNA5S9
LOC124902961 (428bp us/300bp wd)	chr12	71119001	71119300	300	1,416667	0,04761905	3,300097	0,006570039	upstream	428	LOC124902961
TAF11L8 (259bp us/500bp wd)	chr5	17590201	17590700	500	2,15	0,34285714	2,784721	0,002905188	overlapEnd	259	TAF11L8
PRR20E (1296bp us/100bp wd)	chr13	57165801	57165900	100	1,625	0,14285714	2,746461	0,011649799	upstream	1296	PRR20E
LOC105376219 (1261bp us/100bp wd)	chr9	111286201	111286300	100	1,25	0,14285714	2,700805	0,008598402	upstream	1261	LOC105376219
THOC3-AS1 (422bp us/200bp wd)	chr5	175971501	175971700	200	1,1875	0,14285714	2,625893	0,002922794	upstream	422	THOC3-AS1
USP17L22 (181bp us/200bp wd)	chr4	9267801	9268000	200	1,5	0,21428571	2,547885	0,016052	inside	181	USP17L22
RNA5S13 (1186bp us/400bp wd)	chr1	228638401	228638800	400	2,1875	0,32142857	2,540248	0,009993668	upstream	1186	RNA5S13
PRR20D (1331bp us/100bp wd)	chr13	57159201	57159300	100	1,375	0,14285714	2,529464	0,022778516	upstream	1331	PRR20D
HASPIN (1702bp us/100bp wd)	chr17	3722101	3722200	100	1,375	0,14285714	2,517891	0,012363427	upstream	1702	HASPIN
LOC105378733 (1092bp us/100bp wd)	chr1	53438401	53438500	100	1,25	0,14285714	2,504817	0,027949363	upstream	1092	LOC105378733
USP17L20 (276bp us/100bp wd)	chr4	9258401	9258500	100	1,125	0,14285714	2,499592	0,032956499	inside	276	USP17L20
LOC103171574 (204bp us/300bp wd)	chr15	84420901	84421200	300	1,208333	0,14285714	2,483158	0,020509413	upstream	204	LOC103171574
NUP98 (1308bp us/100bp wd)	chr11	3799101	3799200	100	1,125	0,14285714	2,419968	0,024441867	upstream	1308	NUP98
EIF3M (497bp us/100bp wd)	chr11	32583201	32583300	100	1,125	0,14285714	2,418729	0,021473738	upstream	497	EIF3M
NUP98 (1008bp us/100bp wd)	chr11	3798801	3798900	100	1,125	0,14285714	2,418446	0,021473738	upstream	1008	NUP98
NOTCH2NLB (1340bp us/200bp wd)	chr1	148713901	148714100	200	1,125	0,14285714	2,39051	0,021206011	upstream	1340	NOTCH2NLB
RNA5S10 (1292bp us/400bp wd)	chr1	228631801	228632200	400	1,84375	0,32142857	2,359941	0,029122337	upstream	1292	RNA5S10
LOC105376219 (1461bp us/200bp wd)	chr9	111286401	111286600	200	1,5	0,28571429	2,326604	0,035288456	upstream	1461	LOC105376219
KIF22 (1026bp us/100bp wd)	chr16	29789601	29789700	100	1,125	0,14285714	2,3091	0,036412937	upstream	1026	KIF22
DNASE1L2 (915bp us/100bp wd)	chr16	2234801	2234900	100	1,125	0,14285714	2,309065	0,036412937	upstream	915	DNASE1L2
ECHS1 (1646bp us/100bp wd)	chr10	133375001	133375100	100	1,125	0,14285714	2,30077	0,043029812	upstream	1646	ECHS1
LOC124902883 (0bp us/200bp wd)	chr12	14286101	14286300	200	1,125	0,14285714	2,277548	0,036930213	overlapStart	0	LOC124902883
NCOR1P4 (172bp us/100bp wd)	chr21	9530101	9530200	100	1,125	0,14285714	2,268431	0,036553448	inside	172	NCOR1P4
LOC105379514 (163bp us/100bp wd)	chr21	9530101	9530200	100	1,125	0,14285714	2,268431	0,036553448	inside	163	LOC105379514
LOC105373346 (527bp us/100bp wd)	chr2	314901	315000	100	1,125	0,14285714	2,249667	0,048246232	upstream	527	LOC105373346
TAF11L4 (0bp us/500bp wd)	chr5	17521501	17522000	500	3,175	0,88571429	2,194808	0,005394798	overlapStart	0	TAF11L4
POMT1 (688bp us/100bp wd)	chr9	131502001	131502100	100	1,375	0,28571429	2,187154	0,046046271	upstream	688	POMT1
RNA5S6 (1735bp us/100bp wd)	chr1	228623301	228623400	100	1,5	0,28571429	2,183819	0,035745011	upstream	1735	RNA5S6
FRG1EP (176bp us/100bp wd)	chr20	29497301	29497400	100	1,375	0,28571429	2,172424	0,025204087	inside	176	FRG1EP
DCP1A (1314bp us/100bp wd)	chr3	53348901	53349000	100	1,5	0,28571429	2,167087	0,019616767	upstream	1314	DCP1A
LOC105372324 (304bp us/200bp wd)	chr19	21866601	21866800	200	1,1875	0,28571429	2,145314	0,030286061	upstream	304	LOC105372324
LOC105373447 (351bp us/100bp wd)	chr2	17372801	17372900	100	1,625	0,28571429	2,133027	0,038907466	upstream	351	LOC105373447
TAF11L8 (1440bp us/100bp wd)	chr5	17592401	17592500	100	2,125	0,57142857	2,120553	0,00132947	upstream	1440	TAF11L8
TAF11L10 (672bp us/1000bp wd)	chr5	17598501	17599500	1000	2,8625	0,88571429	2,118517	0,007225389	upstream	672	TAF11L10
LOC124904145 (1919bp us/100bp wd)	chr17	43292401	43292500	100	1,625	0,28571429	2,115698	0,035550282	upstream	1919	LOC124904145
RPGRIP1 (1882bp us/200bp wd)	chr14	21278001	21278200	200	1,4375	0,28571429	2,111742	0,036668435	upstream	1882	RPGRIP1
LINC01635 (285bp us/300bp wd)	chr1	22026501	22026800	300	1,208333	0,23809524	2,087582	0,030091977	upstream	285	LINC01635
USP17L13 (1795bp us/100bp wd)	chr4	9223001	9223100	100	1,25	0,28571429	2,059301	0,024496822	upstream	1795	USP17L13
MIR3115 (1504bp us/100bp wd)	chr1	23042701	23042800	100	1,375	0,28571429	2,053329	0,047370642	upstream	1504	MIR3115
RNA5S8 (1073bp us/100bp wd)	chr1	228627101	228627200	100	1,375	0,28571429	2,023568	0,045690769	upstream	1073	RNA5S8
TAF11L5 (1034bp us/400bp wd)	chr5	17523801	17524200	400	2,34375	0,71428571	2,021565	0,008133547	upstream	1034	TAF11L5
FRG1EP (0bp us/400bp wd)	chr20	29497501	29497900	400	1,5625	0,39285714	2,019508	0,012301047	overlapStart	0	FRG1EP
KEAP1 (742bp us/200bp wd)	chr19	10504301	10504500	200	1,25	0,42857143	1,99639	0,023080659	upstream	742	KEAP1

RNA5S8 (1273bp us/300bp wd)	chr1	228627301	228627600	300	2,041667	0,47619048	1,987317	0,040624851	upstream	1273	RNA5S8
MBD3L2 (120bp us/300bp wd)	chr19	7048901	7049200	300	1,791667	0,57142857	1,955612	0,026931347	upstream	120	MBD3L2
RNA5S15 (1614bp us/100bp wd)	chr1	228643301	228643400	100	1,25	0,28571429	1,948676	0,045265158	upstream	1614	RNA5S15
TAF11L3 (0bp us/1100bp wd)	chr5	17517901	17519000	1100	3,272727	1,07792208	1,943526	0,013485239	includeFeature	0	TAF11L3
DCP1A (1614bp us/100bp wd)	chr3	53349201	53349300	100	1,75	0,42857143	1,936472	0,03621088	upstream	1614	DCP1A
USP17L30 (1928bp us/300bp wd)	chr4	9360901	9361200	300	2,25	0,71428571	1,919535	0,02364146	upstream	1928	USP17L30
USP17L25 (1310bp us/300bp wd)	chr4	9328301	9328600	300	1,875	0,66666667	1,918558	0,038599955	upstream	1310	USP17L25
TAF11L7 (157bp us/600bp wd)	chr5	17585001	17585600	600	5,416667	2,0952381	1,8788	0,013723515	overlapEnd	157	TAF11L7
TAF11L7 (342bp us/200bp wd)	chr5	17586101	17586300	200	1,5625	0,5	1,830349	0,043052368	upstream	342	TAF11L7
TAF11L5 (165bp us/100bp wd)	chr5	17525401	17525500	100	4	1,42857143	1,828173	0,007190641	inside	165	TAF11L5
MIR4477B (26bp us/100bp wd)	chr9	63819601	63819700	100	1	0,28571429	1,810943	0,045707951	overlapEnd	26	MIR4477B
DDX11L5 (0bp us/100bp wd)	chr9	10901	11000	100	1,375	0,42857143	1,798942	0,028506459	overlapStart	0	DDX11L5
RCC1L (1072bp us/300bp wd)	chr7	75075301	75075600	300	2,125	0,66666667	1,786534	0,029314054	upstream	1072	RCC1L
XKR8 (587bp us/100bp wd)	chr1	27958901	27959000	100	1,125	0,28571429	1,786205	0,043338602	upstream	587	XKR8
TAF11L8 (1240bp us/100bp wd)	chr5	17592201	17592300	100	1,625	0,57142857	1,748435	0,0112586	upstream	1240	TAF11L8
TAF11L4 (1300bp us/100bp wd)	chr5	17520401	17520500	100	1,625	0,57142857	1,742656	0,036611372	upstream	1300	TAF11L4
TAF11L6 (668bp us/100bp wd)	chr5	17527901	17528000	100	2,625	1	1,721012	0,013939083	upstream	668	TAF11L6
LOC124900298 (460bp us/100bp wd)	chr10	36438101	36438200	100	1,125	0,28571429	1,718085	0,041909174	upstream	460	LOC124900298
FAM230F (276bp us/100bp wd)	chr22	18896401	18896500	100	1,625	0,57142857	1,701815	0,033913302	inside	276	FAM230F
LOC124904138 (1881bp us/100bp wd)	chr17	43253901	43254000	100	1,625	0,42857143	1,699107	0,043811641	upstream	1881	LOC124904138
USP17L15 (0bp us/400bp wd)	chr4	9234201	9234600	400	3,59375	1,35714286	1,690626	0,014721521	overlapStart	0	USP17L15
TAF11L9 (93bp us/300bp wd)	chr5	17594001	17594300	300	4,5	1,9047619	1,678021	0,021693879	inside	93	TAF11L9
ZNF785 (1031bp us/300bp wd)	chr16	30586801	30587100	300	2,333333	0,71428571	1,67139	0,022121224	upstream	1031	ZNF785
USP17L20 (1523bp us/100bp wd)	chr4	9256501	9256600	100	2	0,71428571	1,651569	0,045554739	upstream	1523	USP17L20
TAF11L6 (1168bp us/300bp wd)	chr5	17527201	17527500	300	2,541667	1,28571429	1,630582	0,0325646	upstream	1168	TAF11L6
TAF11L10 (0bp us/600bp wd)	chr5	17597401	17598000	600	4	1,80952381	1,60261	0,030429488	overlapStart	0	TAF11L10
TAF11L9 (906bp us/200bp wd)	chr5	17595301	17595500	200	3,0625	1,5	1,523337	0,021101079	upstream	906	TAF11L9
TAF11L6 (1968bp us/400bp wd)	chr5	17526301	17526700	400	5,25	2,92857143	1,382828	0,020820399	upstream	1968	TAF11L6
TAF11L5 (1734bp us/100bp wd)	chr5	17523401	17523500	100	4	2,14285714	1,308537	0,02806301	upstream	1734	TAF11L5
TAF11L9 (1506bp us/300bp wd)	chr5	17595901	17596200	300	4,291667	2,42857143	1,267022	0,030402118	upstream	1506	TAF11L9
LOC124901971 (1457bp us/100bp wd)	chr8	85824701	85824800	100	0,75	3,14285714	-1,760042	0,015936117	upstream	1457	LOC124901971
TENM2-AS3 (1431bp us/100bp wd)	chr5	167954901	167955000	100	0,125	1,28571429	-2,17901	0,028607915	upstream	1431	TENM2-AS3
LINC00396 (884bp us/400bp wd)	chr13	110052001	110052400	400	0,125	1,78571429	-2,86594	0,007138272	upstream	884	LINC00396
RNA5S7 (1015bp us/300bp wd)	chr1	228624801	228625100	300	1,333333	0	3,613971	0,026555258	upstream	1015	RNA5S7
REPIN1-AS1 (289bp us/100bp wd)	chr7	150372201	150372300	100	1,25	0	3,608477	0,002563601	inside	289	REPIN1-AS1
FOXP1-AS1 (1758bp us/100bp wd)	chr3	71287901	71288000	100	1,25	0	3,564032	0,005287121	upstream	1758	FOXP1-AS1
RNA5S8 (1973bp us/400bp wd)	chr1	228628001	228628400	400	2,25	0,17857143	3,386301	0,008889388	upstream	1973	RNA5S8
RNA5S9 (0bp us/400bp wd)	chr1	228628001	228628400	400	2,25	0,17857143	3,386301	0,008889388	includeFeature	0	RNA5S9
LOC124902961 (428bp us/300bp wd)	chr12	71119001	71119300	300	1,416667	0,04761905	3,300097	0,006570039	upstream	428	LOC124902961
TAF11L8 (259bp us/500bp wd)	chr5	17590201	17590700	500	2,15	0,34285714	2,784721	0,002905188	overlapEnd	259	TAF11L8
PRR20E (1296bp us/100bp wd)	chr13	57165801	57165900	100	1,625	0,14285714	2,746461	0,011649799	upstream	1296	PRR20E
LOC105376219 (1261bp us/100bp wd)	chr9	111286201	111286300	100	1,25	0,14285714	2,700805	0,008598402	upstream	1261	LOC105376219
THOC3-AS1 (422bp us/200bp wd)	chr5	175971501	175971700	200	1,1875	0,14285714	2,625893	0,002922794	upstream	422	THOC3-AS1
USP17L22 (181bp us/200bp wd)	chr4	9267801	9268000	200	1,5	0,21428571	2,547885	0,016052	inside	181	USP17L22
RNA5S13 (1186bp us/400bp wd)	chr1	228638401	228638800	400	2,1875	0,32142857	2,540248	0,009993668	upstream	1186	RNA5S13
PRR20D (1331bp us/100bp wd)	chr13	57159201	57159300	100	1,375	0,14285714	2,529464	0,022778516	upstream	1331	PRR20D
HASPIN (1702bp us/100bp wd)	chr17	3722101	3722200	100	1,375	0,14285714	2,517891	0,012363427	upstream	1702	HASPIN
LOC105378733 (1092bp us/100bp wd)	chr1	53438401	53438500	100	1,25	0,14285714	2,504817	0,027949363	upstream	1092	LOC105378733
USP17L20 (276bp us/100bp wd)	chr4	9258401	9258500	100	1,125	0,14285714	2,499592	0,032956499	inside	276	USP17L20
LOC103171574 (204bp us/300bp wd)	chr15	84420901	84421200	300	1,208333	0,14285714	2,483158	0,020509413	upstream	204	LOC103171574
NUP98 (1308bp us/100bp wd)	chr11	3799101	3799200	100	1,125	0,14285714	2,419968	0,024441867	upstream	1308	NUP98
EIF3M (497bp us/100bp wd)	chr11	32583201	32583300	100	1,125	0,14285714	2,418729	0,021473738	upstream	497	EIF3M
NUP98 (1008bp us/100bp wd)	chr11	3798801	3798900	100	1,125	0,14285714	2,418446	0,021473738	upstream	1008	NUP98
NOTCH2NLB (1340bp us/200bp wd)	chr1	148713901	148714100	200	1,125	0,14285714	2,39051	0,021206011	upstream	1340	NOTCH2NLB

Summary of differentially methylated regions (DMRs), including mean methylation levels for the low- and high-UPF groups ("Low UPF.counts.mean" and "High UPF.counts.mean"), log_2 fold-change values ("edgeR.logFC"), and nominal p-values ("edgeR.p.value"). Regions are ranked from highest to lowest fold-change for clarity and transparency.
